# *Fusobacterium nucleatum* Load Correlates with *KRAS* Mutation and Sessile Serrated Pathogenesis in Colorectal Adenocarcinoma

**DOI:** 10.1158/2767-9764.CRC-23-0179

**Published:** 2023-09-26

**Authors:** Koki Takeda, Minoru Koi, Yoshiki Okita, Sija Sajibu, Temitope O. Keku, John M. Carethers

**Affiliations:** 1 Division of Gastroenterology and Hepatology, Department of Internal Medicine and Rogel Cancer Center, University of Michigan, Ann Arbor, Michigan.; 2 Divsion of Gastroenterology and Hepatology, Department of Medicine and Moores Cancer Center, University of California San Diego, San Diego, California.; 3 Department of Gastrointestinal and Pediatric Surgery, Graduate School of Medicine, Mie University, Mie, Japan.; 4 Institute of Molecular Cancer Research, University of Zurich, Zurich, Switzerland.; 5 Division of Gastroenterology and Hepatology, Departments of Medicine and Nutrition, University of North Carolina at Chapel Hill, Chapel Hill, North Carolina.

## Abstract

*Fusobacterium nucleatum* (*Fn*) has been frequently detected in colorectal cancer. A high load of *Fn* has been associated with subtypes of colorectal cancers, located in the proximal colon, exhibiting microsatellite instability-high (MSI-H), *MLH1* promoter hypermethylation, the *CpG* island hypermethylation phenotype-high, or *BRAF* mutation in some studies. Although these features characterize the sessile serrated pathway (SSP) of colon cancers, other studies have shown that *Fn* infection is associated with *KRAS* mutations mainly characteristic of non-serrated neoplasia. It is also not clear at what point the association of *Fn* infection with these genomic alterations is established during colorectal carcinogenesis. Here we show that MSI-H, *MLH1* hypermethylation, *BRAF* mutation or *KRAS* mutations were independently associated with *Fn* infection in colorectal cancer. On the other hand, increasing *Fn* copy number in tissues was associated with increased probability to exhibit MSI-H, *MLH1* hypermethylation or *BRAF* mutations but not *KRAS* mutations in colorectal cancer. We also show that *Fn* load was significantly less than that of colorectal cancer and no association was detected between *BRAF/KRAS* mutations or *MLH1* hypermethylation and *Fn* infection in adenomas. Our combined data suggest that increasing loads of *Fn* during and/or after adenomacarcinoma transition might promote SSP but not *KRAS*-driven colorectal carcinogenesis. Alternatively, *Fn* preferentially colonizes colorectal cancers with SSP and *KRAS* mutations but can expand more in colorectal cancers with SSP.

**Significance:**

The authors demonstrated that *Fn* is enriched in colorectal cancers exhibiting the SSP phenotype, and in colorectal cancers carrying *KRAS* mutations. *Fn* infection should be considered as a candidate risk factor specific to colorectal cancers with the SSP phenotype and with *KRAS* mutations.

## Introduction

Colorectal cancer is the third most common cancer in incidence and is the second cause of cancer death worldwide. In 2020, it was estimated that 1.9 million new cases would be diagnosed, and 935,000 persons would experience death (1). Despite increased use of screening and improvements in treatment, the burden of this disease is still high (2). Attributable risk for colorectal cancer includes unmodifiable factors such as sex, age, race, and inherited gene mutations, and modifiable factors such as smoking, unhealthy diet, excess alcohol intake, obesity, and lack of physical activity (3). In the United States, more than half of all colorectal cancer cases are attributed to modifiable factors (2).

Because the discovery of abundant presence of *Fusobacterium nucleatum* (*Fn*) in colorectal cancers in 2012, evidence that the makeup of the gut microbiome contributes as a risk factor for colorectal cancer and influences the initiation and progression of colorectal cancer has been accumulating (4–6). Several bacterial species including *Fn*, Enterotoxigenic *Bacteroides fragilis* (*ETBF*), and colibactin-producing *Escherichia coli* are associated with colorectal cancer in epidemiologic studies, found to be enriched in colorectal cancer tissues, and facilitate colorectal tumors in preclinical models (6, 7). Among these species, *Fn* has been detected in most colorectal adenoma/carcinoma tissues examined by studies using 16s rRNA sequencing or metagenomic sequencing (8). Particularly, *Fn* infection is associated with a subgroup of colorectal cancers that are located on the right side of the colon, exhibit the CpG island methylation phenotype (CIMP)-high, and exhibit high levels of microsatellite instability (MSI-H; refs. 9, 10). Recently, by analyzing the sequence datasets from The Cancer Genome Atlas (TCGA) and the European Genome-Phenome Archive (11), Ternes and colleagues reported that *Fn* infection is associated with colorectal cancer belonging to consensus molecular subtypes (CMS) 1 and 3. While the data reported by Ternes and colleagues agree with previous results where *Fn* infection was associated with MSI-H colorectal cancer that consists of 74% of CMS1 colorectal cancer in TCGA cohort, the association of *Fn* infection with CMS3 colorectal cancer has not been reported previously (11–13). CMS3 colorectal cancers are characterized as being metabolically deregulated and enriched in *KRAS* mutations (70% of CMS3 in TCGA cohort; ref. 12). Although several studies demonstrated that MSI-H, *BRAF* mutation, *MLH1* hypermethylation, or CIMP-high, characteristics shared with CMS1 colorectal cancer were associated with high levels of *Fn* infection, none of these studies showed any association between *Fn* infection and *KRAS* mutations (14–20). On the other hand, other studies have shown that *Fn* infection is associated with *KRAS* mutations in colorectal cancer (21–24). These discrepancies among previous studies could be due to the difference in methods to detect and quantitate *Fn*, and/or difference in cut-off levels to discriminate *Fn*-positive and *Fn*-negative (21).

We previously reported that *Fn* directly caused DNA damage in infected tissues and its infection was enriched in two subgroups of colorectal cancers, one exhibiting MSI-H and another exhibiting low levels of microsatellite instability (MSI-L) and elevated microsatellite alterations in selected tetranucleotide repeats (EMAST; L/E; ref. 10). We have shown that L/E exhibited by colorectal cancer is induced by dysfunction of the mismatch repair protein, MSH3, and is associated with tissue inflammation (25, 26). These results suggest that *Fn* infection might contribute to colorectal carcinogenesis not only by affecting the cellular DNA but also the tumor microenvironment. Regarding the status of *BRAF* mutations, *MLH1* hypermethylation and *KRAS* mutations and their relationship to *Fn* infection, there are conflicting results exists among previous studies (14–22). Because clinical treatment of *KRAS*-mutated colorectal cancer is different from *KRAS* wild-type colorectal cancer, it is important to determine whether this group of colorectal cancers is associated with *Fn* infection that may negatively affect prognosis and the efficacy of chemotherapy (23, 24, 27). In this study, we aimed to determine whether and how *KRAS* mutations are associated with *Fn* infection compared with the association of *BRAF* mutation, *MLH1* hypermethylation and MSI-H with *Fn* using a previously characterized colorectal cancer cohort. We also aimed to determine whether *KRAS*, *BRAF* mutations, and/or *MLH1* hypermethylation is associated with *Fn* infection in colon adenomas.

## Materials and Methods

### Colorectal Cancer and Adenoma Tissue Cohorts

The colorectal cancer cohort used in this study has been described previously (10) and consists of 91 unselected patients with cancers of rectal, sigmoid, or rectosigmoid junction who participated in the North Carolina Colon Cancer Study-Phase II [(NCCCS II) NC Rectal Cancer Study (IRB#99-0933)]. Patients were 40 to 79 years of age, resided in central North Carolina and diagnosed between 2001 and 2006 (28). An additional 213 patients with unselected sporadic colorectal cancer were obtained from the North Carolina site for the Cancer Care Outcomes Research and Surveillance consortium (CanCORS). CanCORS was a population-based prospective, case-only, multisite observational study of patients with colorectal and lung cancer (29). Those patients were at least 21 years of age, residents of central North Carolina, and diagnosed between 2003 and 2006. The study was approved by the University of North Carolina's Institutional Review Board (IRB# 04-0860). All data associated with the 304 total cases of colorectal cancer are presented in Supplementary Table S1. Thirty-two adenoma cases including sessile serrated adenoma (SSA, 10 cases) and tubular adenoma (TA)/tubular-villous adenoma (TVA; 22 cases) were used in this study and have been described previously (30). Fresh adenomas were collected during colonoscopies performed between 2014 and 2017 at Cremona Hospital (Italy) or Zurich Triemli Hospital (Switzerland) with approval of both hospitals’ research ethics committees, and DNA was extracted with AllPrep Mini Kit (QIAGEN). Tissues were histologically classified according to World Health Organization criteria. All data associated with the 32 cases of adenoma/polyp are shown in Supplementary Table S2. All tissues in each of the above cohorts were obtained via written informed consent from patients under IRB approval and all studies were conducted in accordance within recognized international ethical guidelines.

### MSI/EMAST Assay

Paired normal and tumor genomic DNA from all colorectal cancers were prepared from formalin-fixed paraffin-embedded (FFPE) tissues using the QIAamp DNA FFPE Tissue Kit (QIAGEN). The MSI/EMAST assay was carried out as described previously (31). Briefly, the assay consists of 14 microsatellite markers in four reactions capable of determining MSI-H, MSI-L, EMAST, and microsatellite stable (MSS) simultaneously. The markers include two mononucleotide (BAT25 and BAT26), five dinucleotide (D2S123, D5S346, D17S250, D18S64, and D18S69), and seven tetranucleotide microsatellite sequences (D9S242, D20S82, D20S85, D19S394, D8S321, MYCL1, and RBM47). We defined MSI-H, MSI-L/EMAST (L/E), and MSS as described previously (32).

### Detection and Quantification of *Fn* DNA from Tumor Tissues

The method for detection and quantification of *Fn* DNA in colorectal cancer has been described before (10). Briefly, the absolute quantity of *Fn* DNA and that of tumor DNA in each sample was determined separately by the SYBER green–based standard curve method. The PCR primer set specific to *Fn* was designed to target the *nusG* gene of *Fn* (5) and the primer set specific to the human genome targets the non-gene coding region of human chromosome 9p24 (chr9:242200-2242300; GRCH38/hg38). Each reaction contained 2.5 ng of DNA, 1X Power SYBER Green Master MIX (Applied Biosystems), and 250 nmol/L of each primer and all samples were assayed in duplicate in 10 µL reactions. Amplification and detection of DNA were carried out using the ABI 7900HT Sequence Detection System (Applied Biosystems). The primer sequences for *Fn* and human genome at 9p24 were described before (10). Specificity of PCR products for *Fn* and human 9p24 region was monitored by comparing melting curves generated from reference *Fn* DNA (*Fn* strain VPI4355, ATCC) and genomic DNA from the human colon cancer cell line, DLD1. We also used the TaqMan-based standard curve method for detection and quantification of *Fn* DNA in adenoma/polyp tissues. The PCR primer and FAM probe sequences were as follows: *Fn* forward primer, 5′-GCTTGAAATGGAAGCTACAAGAG; *Fn* reverse primer, 5′-GGATCA GAACCAACTCCTACAA; *Fn-*FAM probe: 5′-AGTAGACCCTCGTGTATG. PCR conditions were 95°C for 10 minutes, followed by 45 cycles of 95°C for 15 seconds and 54°C for 1 minute. The reaction mixture consisted of TaqMan Universal Master Mix (Applied Biosystems), 300 nmol/L forward, 900 nmol/L reverse primers, 250 nmol/L *Fn*-FAM probe and approximately 50 ng of template DNA. For quantifying tumor DNA in adenoma samples, we used the SYBER green–based standard curve method as used for colorectal cancer samples.

Sensitivity of SYBER green–based standard curve method and TaqMan-based standard curve method to detect *Fn* DNA was examined as follows: We generated a standard curve using the same amount of template *Fn* genomic DNA (4-fold dilution starting from 1,000 ng/reaction) isolated from the *Fn* strain VPI4355 (ATCC) for each assay (Supplementary Fig. S1). In SYBER green–based assay, the slope of the reaction was −3.272 and amplification efficiency was 102% whereas the slope and amplification efficiency of the TaqMan-based assay was −3.62 and 89%, respectively. There was a about 2-fold difference (average: 2.29) in CT value at each DNA dilution point between SYBER green–based and TaqMan-based assay, indicating that TaqMan assay is approximately four times less sensitive for detecting *Fn* DNA. To compensate the different sensitivity, we included 10 times more adenoma DNA (50 ng) in TaqMan PCR reaction compared with colorectal cancer DNA (5 ng) in SYBER green assay. *Fn* DNA content in each sample was determined by the following formula: absolute *Fn* DNA in picogram/absolute tumor genomic DNA in nanograms. On the basis of the fact that the total *Fn* genome contains 2,170 kilobase pairs (33), the number of copies of *Fn* per nanogram of tumor DNA was calculated from *Fn* DNA content.

### *KRAS(G12/G13)* and *BRAFV600E* Mutation Detection

Mutational hot spots on *KRAS* codons 12 and 13, and on *BRAF* codon 600 involving valine to glutamic acid were investigated by PCR-direct sequencing. PCR reactions on extracted DNA were performed with the Q5 High-Fidelity master mix (New England Biolaboratories) in a 96-well thermal cycler (Applied Biosystems). The primers used were: *KRAS* forward primer: 5′-GGTACTGGTGGAGTATTTGATAGTG-3′, *KRAS* reverse primer: 5′-ACCTCTATTGTTGGATCATATTCGT-3′, *BRAF* forward primer: 5′-TGCTTGCTCTGATAGGAAAATG-3′, and *BRAF*-reverse primer: 5′-AGTAACTCAGCAGCATCTCAGG-3′.

The PCR products were purified by ExoSap-IT (Applied Biosystems) and sent to an outsource vender (Eurofins) for cycling sequencing.

### *MLH1* Promoter Hypermethylation Detection and MLH1 Immunostaining

The DNA isolated from tumor tissues or cell lines (∼500 ng) was modified with sodium bisulfite using EZ DNA Methylation-Gold Kits (D5005, Zymo Research). Methylation-specific PCR (MSP) was used for detecting promoter methylation of the *hMLH1* locus (34). The sequences of the methylated-specific and unmethylated-specific primer pair and PCR cycling conditions were the same as described previously (34), except that AmpliTaq Gold 360 master mix (Applied Biosystems) was used for amplification. The PCR products were separated on 3% MetaPhor agarose (Lonza), stained with GelRed Nucleic Acid Stain (Biotium), and then visualized with UV illumination using a digital imaging system (ImageQuant LAS 4000, GE Healthcare). As a positive control for the *MLH1* promoter hypermethylation, bisulfite DNA from the human colon cancer cell line RKO was used. As a negative control, bisulfite DNA from SW480 cells was used. If the band intensity of the methylation-specific PCR products was equal to or greater than that of the unmethylated PCR products on the gel image, the sample was defined as methylation-positive. To confirm the results obtained by MSP analysis, MLH1 IHC staining was performed on the samples that were positive for MLH1 MSP. Paraffin-embedded tissues were deparaffinized and rehydrated. After antigen retrieval (121°C for 15 minutes in 0.01 mol/L citrate buffer, (pH 6.0), the tissues were treated overnight at 4°C with anti-human MLH1 mouse antibody (1:400, catalog no.: 550838, BD Biosciences), followed by visualization of the MLH1 signal with ImmPRESS Universal PLUS Polymer Kit (MP-7800, Vector Laboratories)

### Statistical Analysis

All statistical analyses were carried out using the software XLSTAT (Addinsoft). The association between *Fn* infection and each genomic variable, and other variables, including sex, age, tumor location and stage, in colorectal cancer cohort was tested using a logistic regression model with Firth bias correction. The association between *Fn* infection and various variables in adenoma/polyp cohort was tested using Fisher exact test. The Mann–Whitney test and Kruskal–Wallis test were used to determine the difference in the amounts of *Fn* DNA present in different groups of colorectal cancers. The Kaplan–Meier test was used to determine whether *Fn* infection affected patients’ 5-year overall survival (OS) rate. When *P* values were less than 0.05, the difference or association was determined significant.

### Data Availability Statement

The data generated in this study are available upon request from the corresponding author.

## Results

### Characterization of Colorectal Cancer Cohort

We previously examined 304 cases of unselected colorectal cancer derived from North Carolina for determining the relationship between *Fn* infection and molecular subtypes of colorectal cancer including MSI-H, L/E, and MSS (10). Among the 304 cases previously used, five cases (including one case with MSI-H, two cases with L/E, and two cases with MSS) had depleted template DNA. Therefore, these cases were replaced by new cases of MSI-H (one case), L/E (two cases), and MSS (two cases) from the CanCORS cohort. In this revised cohort, 45.1% (137/304) were MSS, 42.4% (129/304) were L/E, 12.5% (38/304) were MSI-H, and 87.5% (266/304) of colorectal cancer non-MSI-H (Table 1; Supplementary Table S1).

**TABLE 1:** Association of *Fn* infection and genomic/clinicopathologic factors in 304 cases of colorectal cancers

			No. of cases (%)		Univariate	
Variables		No. of cases (%)	*Fn*-positive	*Fn*-negative	OR	*P* value^a^
	Total	304	109 (35.9)	195 (64.1)		
MSI L/E MSS status	MSS	137 (45.1)	34 (24.8)	103 (75.2)		
	L/E	129 (42.4)	52 (40.3)	77 (59.7)		
	MSI-H	38 (12.5)	23 (60.5)	15 (39.5)		
	MSS vs. L/E				1.9	**0.01**
	MSS vs. MSI-H				4.34	**<0.001**
	L/E vs. MSI-H				2.28	**0.03**
MSI status	MSI-H	38 (12.5)	23 (60.5)	15 (39.5)		
	Non-MSI-H	266 (87.5)	86 (32.3)	180 (67.7)		
	Non-MSI-H vs. MSI-H				3.12	**0.002**
*BRAF*V600E	Wild	279 (91.8)	95 (34)	184 (66)		
Mutation	Mutated	25 (8.2)	14 (56)	11 (44)		
	Wild vs. Mutated				2.39	**0.04**
*MLH1*	No	279 (91.8)	94 (33.7)	185 (66.3)		
Hypermethylation	Yes	25 (8.2)	15 (60)	10 (40)		
	No vs. Yes				2.84	**0.016**
*KRAS* (G12/G13)	Wild	203 (66.8)	64 (31.7)	138 (68.3)		
Mutations	Mutated	101 (33.2)	45 (44.6)	56 (55.4)		
	Wild vs. Mutated				1.74	**0.02**
Tumor site	Rectum	71 (23.4)	15 (21.1)	56 (78.9)		
	Colon	233 (76.6)	94 (40.3)	139 (59.7)		
	Rectum vs. Colon				2.5	**0.002**
Tumor stage	Local	85 (28)	29 (34)	56 (66)		
	Regional/Distant	173 (56.9)	70 (40)	103 (60)		
	Unknown	46 (15.1)	9 (19.6)	37 (80.4)		
	Local vs. Regional/Distant				1.34	0.27
Age	≤65	150 (49.3)	50 (33.3)	100 (66.7)		
	>65	152 (50)	58 (38.2)	94 (61.8)		
	Unknown	2 (0.7)	1 (50)	1 (50)		
	<65 vs. >65				1.23	0.36
Sex	Female	143 (47)	47 (32.9)	96 (67.1)		
	Male	161 (53)	62 (38.5)	99 (61.5)		
	Female vs. Male				1.28	0.29
Race	White	235 (77.3)	81 (34.5)	154 (65.5)		
	Black	63 (20.7)	25 (39.7)	38 (60.3)		
	Unknown	6 (2)	3 (50)	3 (50)		
	White vs. Black				1.25	0.43

NOTE: *P* values in bold are significant.

^a^ *P* value was determined by univariate logistic regression analysis.

Twenty-five of 304 cases (8.2%) exhibited *BRAFV600E* mutation. *MLH1* hypermethylation was also detected in 8.2% (25/304) of colorectal cancer. A total of 101 of 304 cases (33.2%) showed mutations at *KRAS* G12/G13 (Table 1; Supplementary Table S1). Furthermore, the cohort exhibited the following clinicopathologic characteristics: tumor site (71 rectum and 233 colon cancers); tumor stage (85 localized and 173 regional/distant cancers); patients’ age (150 cases were ≤65 years and 152 cases were >65y); sex (143 female and 161 male); and race (235 White and 63 Black; Table 1).

Among 304 colorectal cancer cases, *Fn* was detected in 109 cases (Supplementary Fig. S2; Table 1). The minimum amounts of *Fn* DNA detected in our colorectal cancer cohort were 0.02 pg (equivalent to one copy of *Fn* genome) per one nanogram of tumor DNA and the maximum amounts were 291 pg (equivalent to 124,467 copies of *Fn*) per one nanogram of tumor DNA, respectively (Supplementary Fig. S2; Supplementary Table S1).

### Association Between *Fn* Infection and *BRAF*/*KRAS* Mutations/*MLH1* Hypermethylation in Colorectal Cancer

As was true for prior results, more colorectal cancer with L/E (L/E-CRC) was infected with *Fn* than colorectal cancer with MSS (MSS-CRC) by univariate logistic regression analysis (OR: 1.9, *P* = 0.01; Table 1). Significantly more cases of colorectal cancer with MSI-H (MSI-H-CRC) were infected with *Fn* compared with MSS-CRC (OR: 4.34, *P* < 0.001) or L/E-CRC (OR: 2.28, *P* = 0.03; Table 1). When all cases were categorized into MSI-H-CRC and colorectal cancer with non-MSI-H (non-MSI-H-CRC), a higher fraction of the MSI-H cases (60.5%: 23/38) were infected with *Fn* compared with that of the non-MSI-H-CRC cases (32.3%: 86/266, OR: 3.12, *P* = 0.002; Table 1). Regarding *BRAF* mutations, 56% (14/25) of colorectal cancer with *BRAF* V660E mutation (*BRAF*V600E-CRC) were infected with *Fn* whereas significantly fewer of the colorectal cancers with wild-type *BRAF* (non-*BRAF*V600E-CRC; 34%: 95/279) were infected with *Fn* (OR: 2.39, *P* = 0.04; Table 1). In regards to *MLH1* promoter hypermethylation, 25 cases of colorectal cancer exhibited hypermethylation. We performed MLH1 IHC staining for 20 out of 25 cases that exhibited *MLH1* promoter hypermethylation and eight cases that were negative for *MLH1* hypermethylation. All 20 colorectal cancer cases with *MLH1* hypermethylation (*MLH1* hypermethylated-CRC) lost MLH1 expression, whereas the eight colorectal cancer cases with unmethylated *MLH1* (non-*MLH1* hypermethylated-CRC) expressed MLH1 (Supplementary Table S1), indicating that *MLH1* hypermethylation determined by our MSP assay accurately reflects silencing of the *MLH1* expression. Sixty percent of cases with *MLH1* hypermethylation (15/25) were infected with *Fn* while a significantly lower percentage (33.7%, 94/279) of colorectal cancer with unmethylated *MLH1* were infected with *Fn* (OR: 2.84, *P* = 0.016; Table 1). Regarding *KRAS G12/G13* mutations, 44.6% (45/101) of colorectal cancer with *KRAS* mutations (*KRAS* mutated-CRC) were infected with *Fn*. Compared with colorectal cancer without *KRAS* mutations (non-*KRAS* mutated-CRC), a significantly higher percentage of cases were infected with *Fn* (OR: 1.74, *P* = 0.02; Tables 1 and 2). Comparing colon with rectum, the colon was more significantly infected with *Fn* (OR: 2.5, *P* = 0.002; Table 1). Other variables including tumor stage, patients’ age, sex, and racial status were not associated with *Fn* infection (Table 1).

**TABLE 2:** Association of *Fn* infection with genomic/clinicopathologic factors (multivariate analysis)

	Multivariable Model 1					Multivariable Model 2					Multivariable Model 3					Multivariable Model 4				
			95% CI					95% CI					95% CI					95% CI		
Variables	Value	OR	Lower	Upper	*P*	Value	OR	Lower	Upper	*P*	Value	OR	Lower	Upper	*P*	Value	OR	Lower	Upper	*P*
*MSI/EMAST Status*																				
MSS vs. L/E	1	2.7	1.2	5.9	**0.017**	—	—	—	—	—	—	—	—	—	—	—	—	—	—	—
MSS vs. MSI-H	1.5	4.6	2	10.5	**<0.001**	—	—	—	—	—	—	—	—	—	—	—	—	—	—	—
L/E vs. MSI-H	0.6	1.72	1.01	2.95	**0.045**	—	—	—	—	—	—	—	—	—	—	—	—	—	—	—
*MSI status*																				
Non-MSI-H vs. MSI-H	—	—	—	—	—	1.2	3.4	1.6	7.3	**0.002**	—	—	—	—	—	—	—	—	—	—
*MLH1*																				
Hypermethylation																				
No vs. Yes	—	—	—	—	—	—	—	—	—	—	1.3	3.5	1.4	8.8	**0.008**	—	—	—	—	—
*BRAF* status																				
Wild vs. Mutated	—	—	—	—	—	—	—	—	—	—	—	—	—	—	—	1	3.2	1.2	8.2	**0.016**
*KRAS* status																				
Wild vs. Mutated	0.6	1.8	1.1	3.1	**0.03**	0.6	1.9	1.1	3.6	**0.02**	0.6	1.9	1.1	3.2	**0.02**	0.6	2	1.1	3.2	**0.018**
*Tumor site*																				
Rectum vs. Colon	0.6	1.9	0.99	3.7	0.05	0.7	2	1.1	33.9	**0.03**	0.8	2.2	1.2	4.2	**0.015**	0.8	2.2	1.2	4.2	**0.014**
*Tumor stage*																				
Local vs. Regional/ Distant	0.7	1.8	1.03	3.3	**0.04**	0.56	1.8	0.99	3.2	0.05	0.57	1.77	1	3.2	**0.05**	0.52	1.68	0.96	3	0.07
*Age*																				
≤65 vs. >65	0.4	1.5	0.9	2.5	0.2	0.4	1.5	0.9	2.6	0.1	0.3	1.36	0.8	2.4	0.2	0.4	1.35	0.8	2.4	0,2
*Sex*																				
Female vs. Male	0.5	1.6	0.98	2.7	0.06	0.5	1.6	0.96	2.6	0.07	0.5	1.6	0.98	2.7	0.06	0.5	1.6	0.96	2.6	0.07
*Race*																				
White vs. Black	0.17	1.3	0.7	2.3	0.7	0.2	1.2	0.7	2.3	0.5	0.2	1.2	0.7	2.2	0.6	0.2	1.2	0.7	2.3	0.5

NOTE: *P* values in bold are significant.

We next determined independent factor(s) associated with *Fn* infection using multivariate logistic regression modeling (Table 2). Variables including MSI-H, *MLH1* hypermethylation, and *BRAF* mutation revealed severe multicollinearity when measured by variance inflation factor (VIF; VIF for MSI-H was 2.69, 6.41 for *MLH1* hypermethylation, and 4.55 for *BRAF* mutation); we constructed four independent models where these three variables did not overlap. In Model 1, MSI-H was associated with *Fn* infection compared with MSS [OR: 4.6, 95% confidence interval (CI): 2.0–10.5, *P* < 0.001] and with L/E (OR: 1.7, 95% CI: 1.01–2.95, *P* = 0.045). *KRAS* mutations (OR: 1.8, 95% CI: 1.1–3.1, *P* = 0.03) and regional/advanced stage (OR: 1.8, 95% CI: 1.03–3.3, *P* = 0.04) were also associated with *Fn* infection, when compared with non-*KRAS* mutation and local stage, respectively (Table 2). Importantly, in all four models, *KRAS* mutation was independently associated with *Fn* infection (Table 2). Finally, *Fn* infection was associated with MSI-H (vs. non-MSI-H, OR: 3.4, 95% CI: 1.6–7.3, *P* = 0.002 in Model 2), *MLH1* hypermethylation (OR: 3.5, 95% CI: 1.4–8.8, *P* = 0.008 in Model 3) or *BRAF* mutation (OR: 3.2, 95% CI: 1.2–8.2, *P* = 0.016 in Model 4; Table 2). Taken together, these results showed that MSI-H, *BRAF* mutations, *MLH1* hypermethylation, and *KRAS* mutations are independently associated with *Fn* infection in colorectal cancer.

### *Fn* Infection in Colorectal Cancer

**FIGURE 1:**
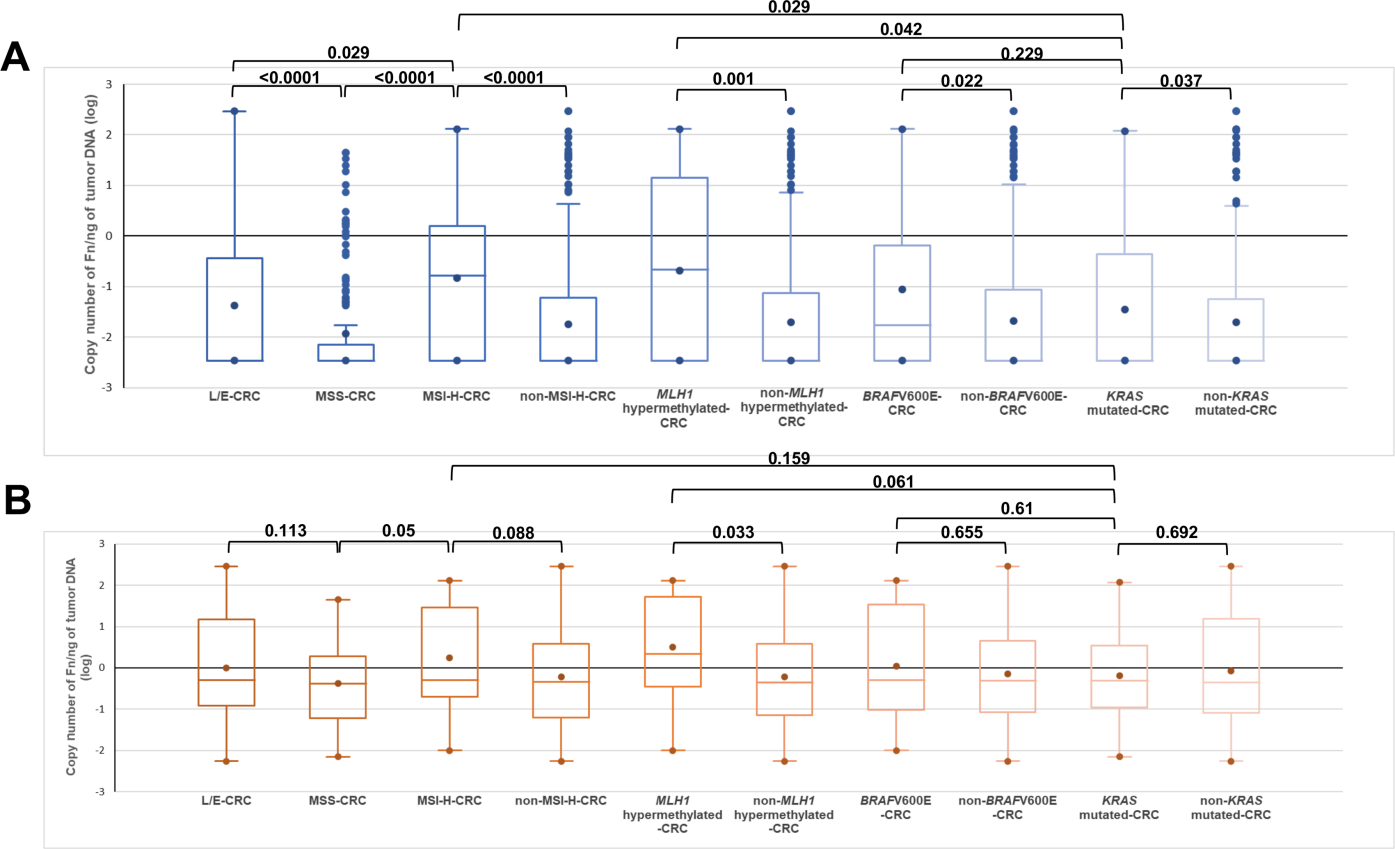
**A,** Comparison of *Fn* loads among subgroups of colorectal cancers. Copy number of *Fn* per nanogram of tumor DNA (log) among L/E-CRC (*n* = 129), MSS-CRC (*n* = 137), MSI-H-CRC (*n* = 38) and non-MSI-H-CRC (*n* = 266), *MLH1* hypermethylated-CRC (*n* = 25) and non-MLH1 hypermethylated-CRC (*n* = 279), *BRAF* V600E-CRC (*n* = 25) and non-*BRAF*V600E-CRC (*n* = 279) and *KRAS* mutated-CRC (*n* = 101) and non-*KRAS* mutated-CRC (*n* = 203) were compared. Data are depicted in each boxplot. The thick horizontal line within each box represents the median copy number of *Fn*. Dots in each column represent maximum (top), mean (middle), and minimum (bottom) copy number of *Fn*. Dots in MSS, non-MSI-H, non-*MLH1* hypermethylated, non-*BRAF*V600E, and non-*KRAS* mutated-CRC column represent outliers. **B,** Comparison of *Fn* loads among subgroups of colorectal cancer that were infected with *Fn*. *Fn* DNA content between *Fn*-infected L/E (*n* = 52), MSS-CRC (*n* = 34), MSI-H-CRC (*n* = 23), non-MSI-H-CRC (*n* = 86), *MLH1* hypermethylated-CRC (*n* = 15), non-MLH1-hypermethylated-CRC (*n* = 94), *BRAF*V600E (*n* = 14) and non-*BRAF*V600E (*n* = 95) and *KRAS* mutated-CRC (*n* = 45) and non-*KRAS* mutated-CRC (*n* = 64) were compared using Kruskal–Wallis test. Each number represents the *P* value for each comparison. A *P* value that is less than 0.05 is considered significant.

To see any difference in *Fn* loads among subgroups of colorectal cancers, we compared the copy number of *Fn* among colorectal cancers with L/E, MSS, MSI-H, non-MSI-H, *MLH1* hypermethylation, non-*MLH1* hypermethylation, *BRAF*V600E, non-*BRAF*V600E, *KRAS* mutation, and non-*KRAS* mutations using the Kruskal–Wallis test (Fig. 1A). The number of *Fn* copies in each sample was converted to log value before comparison. As shown in Fig. 1A, *Fn* loads were the highest in *MLH1* hypermethylated-CRC. MSI-H-CRC and *BRAF*A600E-CRC had the second and third highest *Fn* loads, respectively. *KRAS* mutated-CRC and L/E colorectal cancer had the fourth highest *Fn* loads, and MSS-CRC had the lowest *Fn* loads. As reported previously (10), MSI-H-CRC had higher *Fn* loads than L/E-CRC (*P* = 0.029) and MSS-CRC (*P* < 0.0001), and L/E-CRC had higher *Fn* loads than MSS-CRC (*P* < 0.0001). Here, MSI-H-CRC, *MLH1* hypermutated-CRC, *BRAFV600E*-CRC and *KRAS* mutated-CRC had higher *Fn* loads compared with non-MSI-H-CRC (*P* < 0.0001), non-*MLH1* hypermutated-CRC (*P* = 0.001), non-*BRAFV600E*-CRC (*P* = 0.022) and non-*KRAS* mutated-CRC (*P* = 0.037), respectively (Fig. 1A). Also, the difference in *Fn* loads between *MLH1* hypermethylated-CRC and *KRAS* mutated-CRC (*P* = 0.042) and between MSI-H-CRC and *KRAS* mutated-CRC (*P* = 0.029) was significant.

We then compared *Fn* loads only in *Fn*-infected colorectal cancers from each group. As shown in Fig. 1B, there was no significant difference in the copy number of *Fn* among MSI-H, L/E, and MSS-CRC. There was also no difference in the copy number of *Fn* between (i) MSI-H-CRC and non-MSI-H-CRC (*P* = 0.088); (ii) *BRAF*V600E-CRC and non-*BRAF*V600E-CRC (*P* = 0.655); and (iii) *KRAS* mutated-CRC and non-*KRAS* mutated-CRC (*P* = 0.692). In contrast, *Fn*-infected *MLH1* hypermethylated-CRC contained more *Fn* DNA compared with *Fn*-infected non-*MLH1* hypermutated-CRC (*P* = 0.033; Fig. 1B). Taken together, and as shown in Fig. 1A, *Fn* colonizes in different subgroups of colorectal cancers with differing efficiency but grows at a similar rate once it establishes colonization, except that *Fn* infects with and grows more efficiently in *MLH1* hypermethylated-CRC than in other groups of colorectal cancers as shown in Fig. 1B. Note that *Fn* colonizes less efficiently in *KRAS* mutated-CRC compared with *MLH1* hypermethylated-CRC or MSI-H-CRC; however, it colonizes more efficiently in *KRAS* mutated-CRC compared with non-*KRAS* mutated-CRC (Fig. 1A).We further examined the relationship between each genotype including MSI-H, *MLH1* hypermethylation, *BRAF* mutations, and *KRAS* mutations (response variables) and copy number of *Fn* (explanatory variable) using logistic regression analysis. As shown in Table 3, increasing copy number of *Fn* is associated with a higher probability of colorectal cancer with *MLH1* hypermethylation (OR: 1.56, *P* < 0.0001), MSI-H (OR: 1.5, *P* < 0.0001), and *BRAF*V600E (OR: 1.33, *P* = 0.027), but not with *KRAS* mutations (OR: 1.15, *P* = 0.113) in univariate logistic regression analysis. After adjusting for tumor site and stage, and patients’ age, sex, and race, a higher copy number of *Fn* is independently associated with *MLH1* hypermethylation (OR: 1.46, *P* < 0.0001), MSI-H (OR: 1.5, *P* < 0.0001), slightly with *BRAF*V600E (OR: 1.21, *P* = 0.051) but not with *KRAS* mutations (OR: 1.15, *P* = 0.142; Table 3). These results suggest that interaction between *Fn* and *MLH1* hypermethylated-CRC or MSI-H-CRC, and interaction between *Fn* and *KRAS* mutated-CRC, are biologically different. It could be that increasing the copy number of *Fn* in precursor adenomas with the *BRAF*V600E mutation contributes to promoting hypermethylation of the prompter region of the *MLH1* locus, resulting in MSI-H, leading to transition of adenoma to carcinoma. On the other hand, *Fn* may not be directly involved in *KRAS*-driven carcinogenesis; it may merely colonize more efficiently in *KRAS* mutated-CRC. We also examined the relationship between copy number of *Fn* and MSI-H, L/E, and MSS. It was seen that an increasing number of *Fn* DNA was accompanied by an increased probability of being MSI-H from L/E (OR: 1.35, *P* = 0.004) or from MSS (OR: 1.81, *P* < 0.0001) and was also accompanied by an increased probability of being L/E from MSS (OR: 1.3 *P* = 0.012; Supplementary Table S3). These results suggest that *Fn* infection may contribute to not only inducing MSI-H-CRC through *MLH1* hypermethylation but also inducing L/E-CRC through dysfunction of MSH3 (9). Alternatively, *Fn* more efficiently colonizes MSI-H-CRC than it does L/E-CRC or MSS-CRC, and colonizes better in L/E-CRC than MSS-CRC.

**TABLE 3:** Relationship between copy number of *Fn* and *MLH1* hypermethylation, MSI-H, *BRAF*V600E, and *KRAS* mutations

	*MLH1* hypermethylation			MSI-H			*BRAF*V600E			*KRAS* mutations		
	OR	95% CI	*P* *^a^*	OR	95% CI	*P*	OR	95% CI	*P*	OR	95% CI	*P*
Monovariate												
* * *Fn* DNA Copy number	1.56	1.21–2.0	<0.0001	1.5	1.22–1.88	<0.0001	1.33	1.03–1.72	0.027	1.15	0.97–1.37	0.113
Multivariate^b^												
* * *Fn* DNA Copy number	1.46	1.21–1.77	<0.0001	1.5	1.23–1.82	<0.0001	1.21	0.99–1.48	0.051	1.15	0.93–1.40	0.142

^a^ *P* values were obtained through logistic regression analysis.

^b^Copy number of *Fn* was adjusted for tumor site and stage, patients’ sex, age, and race.

### Prognosis of *Fn*-infected Colorectal Cancer

We then examined whether *Fn* infection has any effects on the 5-year OS rate of patients with colorectal cancer. Although patients with *Fn*-infected colorectal cancer exhibited a shorter 5-year OS rate compared with patients with non–*Fn*-infected colorectal cancer, the difference was not significant (*P* = 0.45, log-rank test; Supplementary Fig. S3A). We also found no significant difference in 5-year OS rate between infected and noninfected patients with colorectal cancer with MSI-H (*P* = 0.24), with *MLH1* hypermethylation (*P* = 0.74), with *BRAF* mutations (*P* = 0.41) and with *KRAS* mutation (*P* = 0.9; Supplementary Fig. S3B–S3E). There results suggest that *Fn* infection might not be a prognostic factor for 5-year OS of patients with colorectal cancer.

### *Fn* Infection and *BRAF*/*KRAS* Mutations/*MLH1* Hypermethylation in Adenomas

We next determined whether *Fn* infection was associated with *BRAF* mutation, *KRAS* mutations or *MLH1* hypermethylation in adenomas/polyps. In this preliminary small study, 32 adenomas examined were dissected from the colon (Supplementary Table S2). Among the 32 adenomas, *Fn* DNA was detected in 14 adenomas, ranging from 0.003 to 7.3 pg per 1 ng of tumor DNA (mean DNA content: 0.28 pg/ng of tumor DNA; Supplementary Table S2). Compared with the amount of *Fn* DNA detected in colorectal cancer tumor tissues (mean DNA content: 5.49 pg/ng of tumor DNA; Supplementary Table S1), the *Fn* DNA detected in adenomas was significantly less (*P* = 0.003) by the Mann–Whitney test. Four of 10 SSA (40%) and 10 of 22 TA/TVA (45%) were positive for *Fn* infection. There was no association between *Fn* infection and adenoma types and histologic dysplasia (Table 4). *BRAF* mutations were found in nine of 10 SSA and one in 10 TA and were not associated with *Fn* infection (Supplementary Table S2; Table 4). *KRAS* mutations were found in one in 10 SSA, four in 14 TA, and four in eight TVA, and there was no association between *KRAS* mutations and *Fn* infection (Supplementary Table S2; Table 4). There was one SSA that was positive for *MLH1* promoter hypermethylation and had no association with *Fn* infection (Table 4). Finally, there was no association of *Fn* infection with age or sex (Table 4). These results suggest that an association between *Fn* infection and *BRAF/KRAS* mutations or *MLH1* hypermethylation may not be established at the adenoma/polyp stage.

**TABLE 4:** Association of *Fn* infection and genomic/clinicopathologic factors in adenomas/polyps

		No. of cases (%)		
Variables		*Fn*-positive	*Fn*-negative	*P* ^a^
Adenoma type^b^	SSA	4 (29)	6 (33)	
	TA/TVA	10 (71)	12 (67)	
	SSA vs. TA/TVA			1
Dysplasia^c^	No Dysplasia	3 (21)	4 (22)	
	LGD	7 (50)	7 (39)	
	HGD/ADC	4 (29)	7 (39)	
	No Dysplasia vs. LGD vs. HGD			0.89
*BRAF* *s*tatus	Wild	10 (71)	12 (67)	
	Mutated	4 (29)	6 (33)	
	Wild vs. Mutated			1
*KRAS* status	Wild	10 (71)	13 (72)	
	Mutated	4 (29)	5 (28)	
	Wild vs. Mutated			1
*MLH1* methylation	No	14 (100)	17 (94)	
	Yes	0	1 (6)	
	No vs. Yes			1
Age	≤67	9 (64)	10 (56)	
	>67	5 (36)	8 (44)	
	≤67 vs. 67>			0.725
Sex	Female	8 (57)	11 (61)	
	Male	6 (43)	7 (39)	
	Female vs. Male			1

^a^ *P* value was determined by Fisher exact test.

^b^SSA: sessile serrated adenoma, TA: tublar adenoma, TVA: tubulovillous adenoma.

^c^LGD: low-grade dysplasia, HLD: high-grade dysplasia.

## Discussion

One of the main findings presented here is that incidence of *Fn* infection is significantly high in colorectal cancer exhibiting not only SSP phenotype including MSI-H, *MLH1* hypermethylation or *BRAF* mutations but also *KRAS* mutations (Tables 1 and 2). A unique finding of our study is that the mode of *Fn* infection differs between colorectal cancers with the SSP phenotype and those with *KRAS* mutations. The quantity of *Fn* in colorectal cancer with MSI-H or *MLH1* hypermethylation is higher than in colorectal cancer with *KRAS* mutations (Fig. 1A). Furthermore, increasing loads of *Fn* are associated with MSI and *MLH1* hypermethylation, suggesting that *Fn* may directly or indirectly cause hypermethylation of the *MLH1* locus, leading to MSI-H (Table 3). On the other hand, *Fn* has a stronger affinity to *KRAS*-mutated colorectal cancer than to non–*KRAS*-mutated colorectal cancer, but the increased load of *Fn* is not associated with *KRAS* mutations. This suggests that *Fn* may not play a role in generating mutations in the *KRAS* gene (Fig. 1A; Table 3). In contrast to many studies, our results did not detect any impact of *Fn* infection on the 5-year OS rate (Supplementary Fig. S3).

In previous studies, there have been conflicting results regarding the effect of *Fn* infection on patients’ prognoses (refs. 17, 19, 20, 21, 35–42; Supplementary Table S4) and on the association of *Fn* infection with various molecular characteristics of colorectal cancer (refs. 10, 14–22, 35–37, 40, 42–46; Supplementary Table S4). To compare our results with these studies, we summarized 22 studies that explored the relationship between *Fn* infection and prognosis or molecular characteristics of colorectal cancer in Supplementary Table S4. As shown in the table, factors including biospecimens [fresh frozen (FF), FFPE, and methacarn], *Fn* detection/quantification methods (TaqMan, SYBAR green, Sequencing), number of cases examined, and difference in comparison (*Fn*-high vs. *Fn*-low/negative or *Fn*-positive versus *Fn*-negative) may have influenced the outcome of each study.

We divided these studies into two groups, A and B. Eleven studies in Group A used the TaqMan-based qPCR assay originally described by Castellarin and colleagues (5, 14–19, 36, 42–45). In this original article, nucleotide sequences of the probe overlapped with that of the PCR forward primer. Later, it was reported by Repass and colleagues that the probe sequence from the original article was incorrect and a different and new correct probe was used in their reproducibility experiments (47). The results obtained in the studies using the incorrect *Fn* probe may deserve further investigation for reproducibility. In contrast, the other 11 studies in Group B used various regimens to detect and quantify *Fn* loads (refs. 10, 20–22, 35, 37–41, 46; Supplementary Table S4). Here, we compare our results further with those of the studies listed in Group B.

It was reported that differences in biospecimen, FF/methacarn-fixed versus FFPE, made a difference in the detection and quantification of *Fn* DNA by PCR. Lee and colleagues showed that *Fn* was detected in 41% of FFPE colorectal cancer samples whereas *Fn* was detected in 100% of matched methacarn-fixed tissues from the same patients. They also showed that *Fn* was detected in 10 of 10 FF tissues examined whereas *Fn* was detected in only 12% of FFPE tissues (40). In our previous study, *Fn* was detected in 75% of FF tissues while it was detected in 38% of FFPE samples (10). These results indicate that FF or methacarn-fixed tissues are superior to FFPE tissues for assessing *Fn* DNA present in the original tissues. As shown in Supplementary Table S4, eight studies used FF tissues, one study used methacarn-fixed tissues while our current study and two other studies used FFPE tissues. Regardless of the various detection methods used, all six studies that examined FF or methacarn-fixed colorectal cancer tissues found a significantly negative impact of high loads of *Fn* on OS and/or recurrence-free survival (RFS) of patients with colorectal cancer (refs. 20, 21, 35, 37, 38, 40; Supplementary Table S4). Although the study using FFPE tissues by Yan that showed a high rate of *Fn* detection and significantly worse impact of high loads of *Fn* on patients’ cancer-specific survival and RFS (39), our study and that of Bundgaard-Nielsen, which examined FFPE colorectal cancer samples, found no impact of *Fn* infection on OS of patients with colorectal cancer (ref. 41; Supplementary Fig. S3). These results agree with those of a study by Kim and colleagues (48) and suggest that tissues with high *Fn* loads that have an impact on the prognoses of patients can be accurately identified by studies using FF or methacarn-fixed tissues or in some studies using FFPE samples if the *Fn* DNA is effectively amplified. In contrast, tissues with high loads of *Fn* may fail to be identified in many studies using FFPE samples such as ours reported here.

As shown by our previous studies, MSI-H was associated with *Fn*-positive samples in two independent colorectal cancer cohorts, one cohort consisting of FF samples from Japan, and another consisting of FFPE samples from the United States (10). Here, *MLH1* hypermethylation and *BRAF* mutations were also significantly associated with *Fn*-positive colorectal cancer samples even though template DNA for *Fn* amplification was isolated from FFPE samples (Tables 1 and 2). These results suggest colorectal cancer with MSI-H, *MLH1* hypermethylation or *BRAF* mutations must be heavily and/or specifically infected with *Fn,* so that reduction in amplifiable *Fn* DNA by FFPE treatment may not change these associations. In accordance with the results obtained from our studies using FFPE samples, two studies using FF tissues showed that high loads of *Fn* are associated with MSI-H (refs. 20, 40; Supplementary Table S4). Although the study by Proença did not see an association between any levels of *Fn* and MSI-H, this could be due to the small size of the cohort (43 cases; ref. 22; Supplementary Table S4). In the study by Wei, high loads of *Fn* were associated with loss of MLH1 expression, likely caused by *MLH1* hypermethylation (37). In the study by Yamaoka, although the association between high loads of *Fn* and *MLH1* hypermethylation was not significant, this could be due to the small number of cases with *MLH1* hypermethylation in this cohort (nine cases among the 100 cases examined; ref. 21). In the study by Shariati, *BRAF* mutations were not associated with *Fn*-positivity; however, the number of *BRAF*-mutated cases were only two cases out of a total of 30 cases examined, leading to statistical insignificance (46). In another study by Kunzmann, lack of association of *BRAF* or *KRAS* mutations with high loads of *Fn* was reported; however, this may be because more than a one-third of the total cases (68 cases among 190 cases) were not examined for *BRAF* and *KRAS* mutations (20). In a study by Flanagan, the *Fn* load between colorectal cancer with *KRAS* mutations and those with non-*KRAS* mutations, as well as colorectal cancer with *BRAF* mutations and those with non-*KRAS* mutations were compared. Although the results showed no difference in quantity of *Fn* between *BRAF*-mutant and non–*BRAF*-mutant colorectal cancer or between *KRAS*-mutant and non–*KRAS*-mutant colorectal cancer, the question of whether high loads of *Fn* were associated with *BRAF* or *KRAS* mutations was not determined (35). Thus, although our results are the first to show a significant association between *Fn* infection and *MLH1* hypermethylation or *BRAF* mutations among studies in Group B, our results need to be confirmed by future studies.

Regarding *KRAS* mutations, our results suggest that *Fn* may specifically infect colorectal cancer with *KRAS* mutations at low levels compared with colorectal cancer with non-*KRAS* mutations. Therefore, reduction in amplifiable *Fn* DNA by FFPE treatment may not change these associations (Fig. 1A). In Group B, three of four studies showed a significant association between *Fn* infection and *KRAS* mutations (20–22, 46). This agreed with our results (Supplementary Table S4). As mentioned above, the study by Kunzmann showed no association between *Fn* infection and *KRAS* mutations. This may be because of its failure to determine the mutation status of a large portion (68 cases) of the cohort (190 cases; ref. 20).

By comparing our results with those of the 11 previous studies listed in Group B, we could conclude that patients with high loads of *Fn* may show a shorter OS rate. The studies using FFPE tissues for *Fn* detection by PCR may fail to show this observation because of loss of amplifiable *Fn* DNA. High levels of *Fn* may infect colorectal cancer with MSI-H and *MLH1* hypermethylation and this association can be detected in FF as well as FFPE colorectal cancer samples. Low levels of *Fn* may specifically infect colorectal cancer with *KRAS* mutations as compared with colorectal cancer with non-*KRAS* mutations and this association can be detected in either FF or FFPE colorectal cancer samples. In support of the association of *Fn* with MSI-H or *KRAS* mutations, although indirect, Ternes and colleagues showed that *Fn* infection is significantly enriched in CMS1 where 74% are MSI-H and in CMS3 where 70% carry *KRAS* mutations (11).

Our small non-cancer study suggests that there is no association between *BRAF*/*KRAS* mutations or *MLH1* promoter hypermethylation and *Fn* infection in adenoma/polyp in contrast to carcinoma. There was also no association between *Fn* infection and adenoma tissue type (SSA vs. TA/TVA). *MLH1* promoter hypermethylation was detected in one *BRAF*-mutated adenoma in our cohort (Table 4). These observations suggest that *Fn* infection may occur regardless of *KRAS/BRAF* mutation status or type of adenomas. Together with the colorectal cancer data presented here, the association of genetic alterations including MSI-H, *BRAF* mutation, and *KRAS* mutations or *MLH1* hypermethylation with *Fn* infection may be established during and/or after adenomacarcinoma transition.

Our results raise the question of why and how *Fn* infection is enriched in colorectal cancer with SSP phenotypes including MSI-H, *MLH1* hypermethylation, and *BRAF* mutations, and, independently, *KRAS* mutations. Compared with carcinoma, *Fn* infection in adenoma/polyps seems random, not selective to *BRAF-* or *KRAS-*mutated cases and is characterized by poor bacterial-enabling growth as indicated by a lower number of *Fn* copies. One possibility is that prolonged *Fn* infection, even at a low level in adenomas, may induce genome-wide hypermethylation of the *CpG* island promoter of tumor suppressor genes (TSG), leading to silencing of the expression of the TSGs. It has been shown that *BRAF* or *KRAS* mutations in initiated cells co-operate with silenced TSGs by promoter hypermethylation to progress toward carcinoma formation (49, 50). Thus, SSA with *BRAF* mutations and TA/TVA with *KRAS* mutations may have a selective advantage for progressing to carcinoma when their TSGs are hypermethylated and inactivated through *Fn* infection. Furthermore, if hypermethylation occurs at the *MLH1* locus, the adenoma will result in MSI-H colorectal cancer (9). In support of this possibility, *Fn* infection has been associated with CIMP not only in colorectal cancer, but also in colon tissues from patients with ulcerative colitis (51). Another study showed that bacteria such as *Fn* and *Hungatello hathewayi* are frequently found in colorectal cancer, are associated with TSG promoter hypermethylation, and upregulate DNMT activity in infected cells (52). If this is the case, *Fn* infection must be associated with two sets of hypermethylated TSGs, one such as *MLH1* is accompanied with *BRAF*V600E and another is accompanied with *KRAS* mutations (53, 54).

Another possibility is that an association of *Fn* infection with the SSP phenotype may be established after tumors progress to carcinoma. In this scenario, *Fn* may have greater affinity for colorectal cancers with SSP than with other colorectal cancers, so that *Fn* may efficiently colonize them. For instance, overexpression of galactose and/or GalNAc on the surface of tumor tissues has been a target of *Fn* infection and colonization through Fap2 (55). There is the possibility that the content of Gal-GalNAc or GalNac on the surface of colorectal cancers with the SSP phenotype is relatively higher than that of other subgroups of colorectal cancers. In support of this scenario, the expression of the Tn antigen (GalNca-Ser/Thr), a precursor of Gal-GalNc, is elevated in human cancer cells with a *BRAF* mutation or in colon tissues from *BRAF*^V600E^−inducible mouse models (56). Also, loss of the GALNT6 protein associated with the SSP phenotype results in the expression of truncated O-glycan on the cell surface, leading to an increase in Tn antigen (57). This increased Gal-GalNac contents on cell surface of SSP tumors may not only attract *Fn* but also provide space for further proliferation of *Fn*. On the other hand, affinity of *Fn* to *KRAS*-mutated adenoma or carcinomas could be explained by *KRAS* mutated tissues’ requirement of a higher number of amino acids to survive and multiply in a nutrient-deficient environment (58). It has been shown that *Fn* produces various metabolites including formate, succinic acid, 2-hydroxybutyrate, and amino acids such as glutamic acid, aspartic acid, glycine, isoleucine, leucine, phenylalanine, and valine *in vitro* when contact with colon cancer tissues, or *in vivo* (11). Thus, presence of *Fn* may be advantageous for survival and growth of *KRAS*-mutated adenoma and/or carcinoma.

Although a small preliminary study, this is the first report showing that there is no association between *Fn* infection and *BRAF* or *KRAS* mutations in colon adenoma/polyp tissues in contrast to colorectal cancer, the small number of samples of adenoma/polyp tissues (32 cases) limited the statistical power of this work. Further study analyzing a cohort with a larger sample size is necessary. Another limitation of this study is that FFPE tissues were used to determine the effect of *Fn* infection on colorectal cancer patients’ OS rate. By comparing ours with other studies, we conclude that the ability to detect and quantify *Fn* in FFPE samples by PCR is limited. Therefore, our results showing a lack of association between *Fn* infection and patients’ OS rate needs to be reevaluated through future study.

In this study, we were not able to determine whether *Fn* infection is associated with a shorter RFS rate; some studies showed that *Fn* infection in tumor tissues is associated with shorter RFS of colorectal cancer patients’ postchemotherapy (27, 37, 39). This phenomenon could be explained by (i) acquired resistance of colon cancer cells to the toxic effects of 5-fluorouracil and/or oxaliplatin (27, 59); (ii) reduced antitumor immune response (60, 61); and/or (iii) increased stemness (11) by *Fn* infection. Our results show that *Fn* infection is enriched in clinically distinctive subgroups of colorectal cancers, MSI-H, and *KRAS*-mutated colorectal cancers. Therefore, it is important to determine whether *Fn* infection is a critical factor that modifies response to chemotherapy and the prognosis of colorectal cancers with MSI-H and *KRAS* mutations.

## Supplementary Material

Supplementary Table S1 Table S1 lists data for 306 cases of colorectal cancers

Supplementary Table S2 Table S2 lists data for 32 cases of adenomas

Supplementary Table S3 Table S3 shows relationship between Fn copy number and genetic pathway in colorectal cancers

Supplementary Table S4 Table S4 summarizes 22 studies and relationship of Fn infection and colorectal cancer

Supplementary Fig S1 Fig S1 compares TaqMan and SYBER Green assays for detecting Fn in tissues

Supplementary Fig S2 Fig S2 shows the distribution of Fn infection in colorectal cancers

Supplementary Fig S3 Fig S3 shows Kaplan-Meier curves for overall survival in colorectal cancer patients
