# Supplementary material for: Fusobacterium nucleatum Load Correlates with KRAS Mutation and Sessile Serrated Pathogenesis in Colorectal Adenocarcinoma
Source: Cancer Res Commun. 2023 Sep 26;3(9):1940–51. doi: 10.1158/2767-9764.CRC-23-0179 (PMC10530411; doi:10.1158/2767-9764.CRC-23-0179)
Supplement: Supplementary Fig S1 — Fig S1 compares TaqMan and SYBER Green assays for detecting Fn in tissues [file crc-23-0179-s05.pdf]

Supplemetary Fig. 1

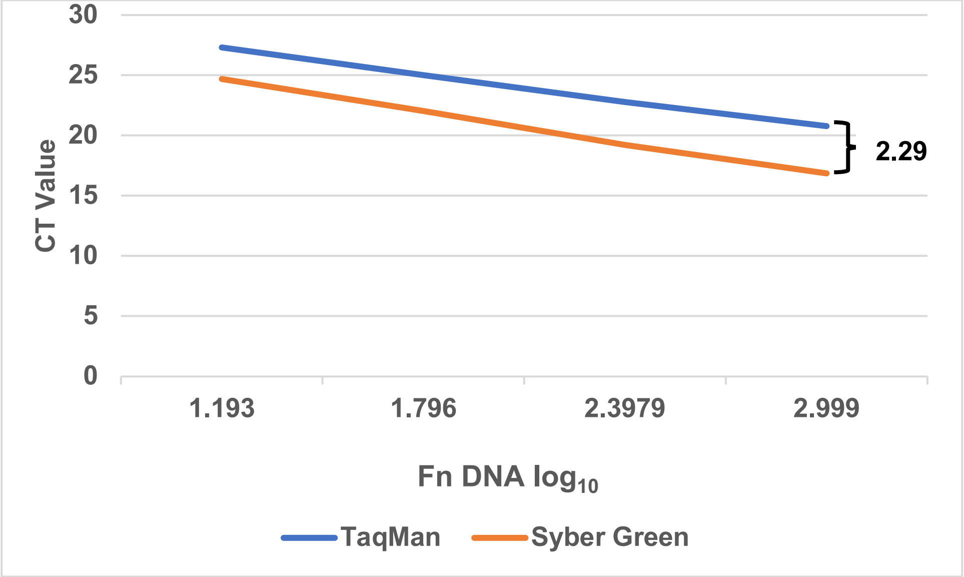

TaqMan assay: Y= 31.56-3.62X, amplification Efficiency: 89%

Syber Green assay: Y= 28.53-3.27X, amplification Efficiency: 102%,

Supplementary Fig. 1: Comparison of sensitivity in detecting *Fn* DNA between TaqMan- and SYBER Green-based Assay. The standard curve was generated using a series of 4-fold diluted *Fn* DNA (1000, 250, 62.5 and 15.625 ng/ul) in triplicate as templates. The TaqMan-based assay yielded a linier relationship between log_{10} value of template DNA concentrations (X) and CT values (Y) presented as Y= 31.56-3.62X, whereas the Syber Green Assy yielded a linier relationship of Y= 28.53-3.27X. The average difference in CT valued between the TaqMan and Syber Green assays was 2.29, indicating that the TaqMan assay is about 4 times less sensitive than the SYBER Green assay.
