# Supplementary material for: Fusobacterium nucleatum Load Correlates with KRAS Mutation and Sessile Serrated Pathogenesis in Colorectal Adenocarcinoma
Source: Cancer Res Commun. 2023 Sep 26;3(9):1940–51. doi: 10.1158/2767-9764.CRC-23-0179 (PMC10530411; doi:10.1158/2767-9764.CRC-23-0179)
Supplement: Supplementary Fig S2 — Fig S2 shows the distribution of Fn infection in colorectal cancers [file crc-23-0179-s06.pdf]

Supplemetary Fig. 2

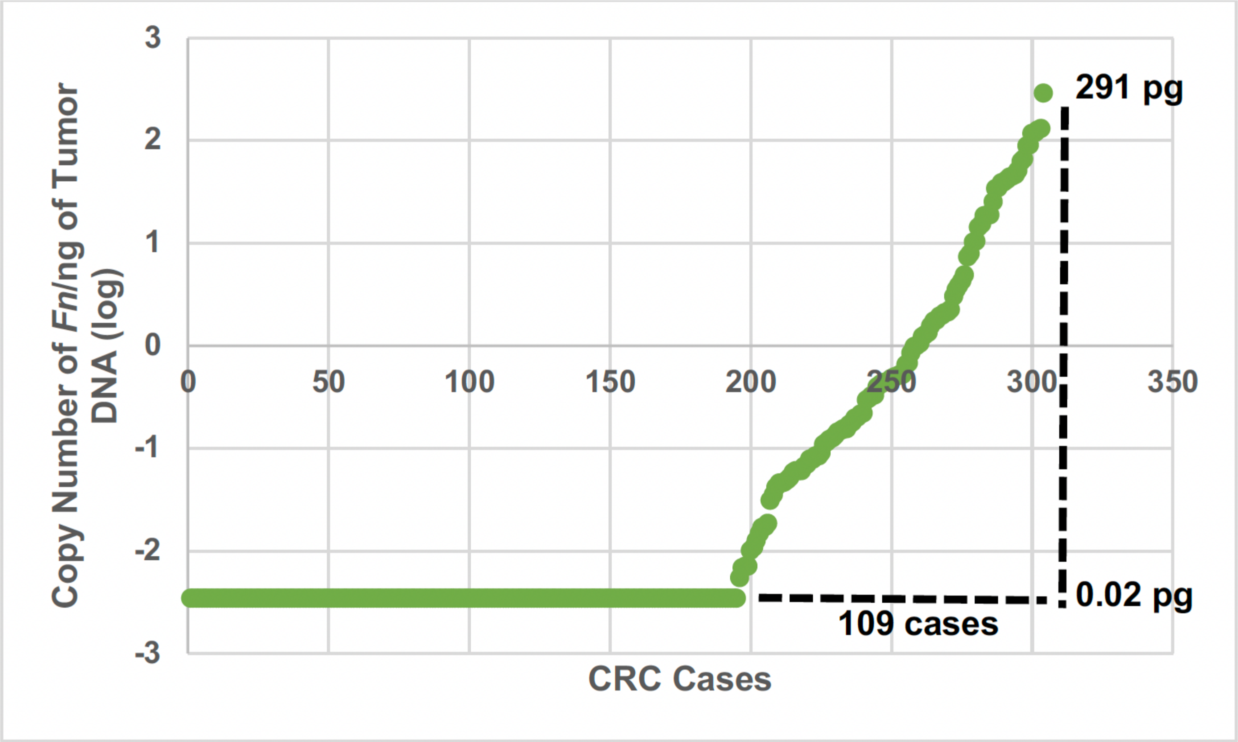

Supplementary Fig. 2: *Fn* infection in CRC. The absolute *Fn* DNA weight was normalized by the absolute tumor DNA weight in each sample. Normalized values were converted to the copy number of *Fn* and to logarithmic scale. Distribution of *Fn* in CRC patients (n=304). The cases were ranked according to the number of copies of *Fn* per nanogram of tumor DNA (log). Each blue dot represents the number of copies of *Fn* in each sample. The horizontal dashed line represents the range of CRC samples that were positive for *Fn* (109 cases). The vertical dashed line indicates the range of number of copies of *Fn* from the minimum (1 copy) to the maximum (124,467 copies) detected.
