# Supplementary material for: Fusobacterium nucleatum Load Correlates with KRAS Mutation and Sessile Serrated Pathogenesis in Colorectal Adenocarcinoma
Source: Cancer Res Commun. 2023 Sep 26;3(9):1940–51. doi: 10.1158/2767-9764.CRC-23-0179 (PMC10530411; doi:10.1158/2767-9764.CRC-23-0179)
Supplement: Supplementary Fig S3 — Fig S3 shows Kaplan-Meier curves for overall survival in colorectal cancer patients [file crc-23-0179-s07.pdf]

Supplementary Fig. 3

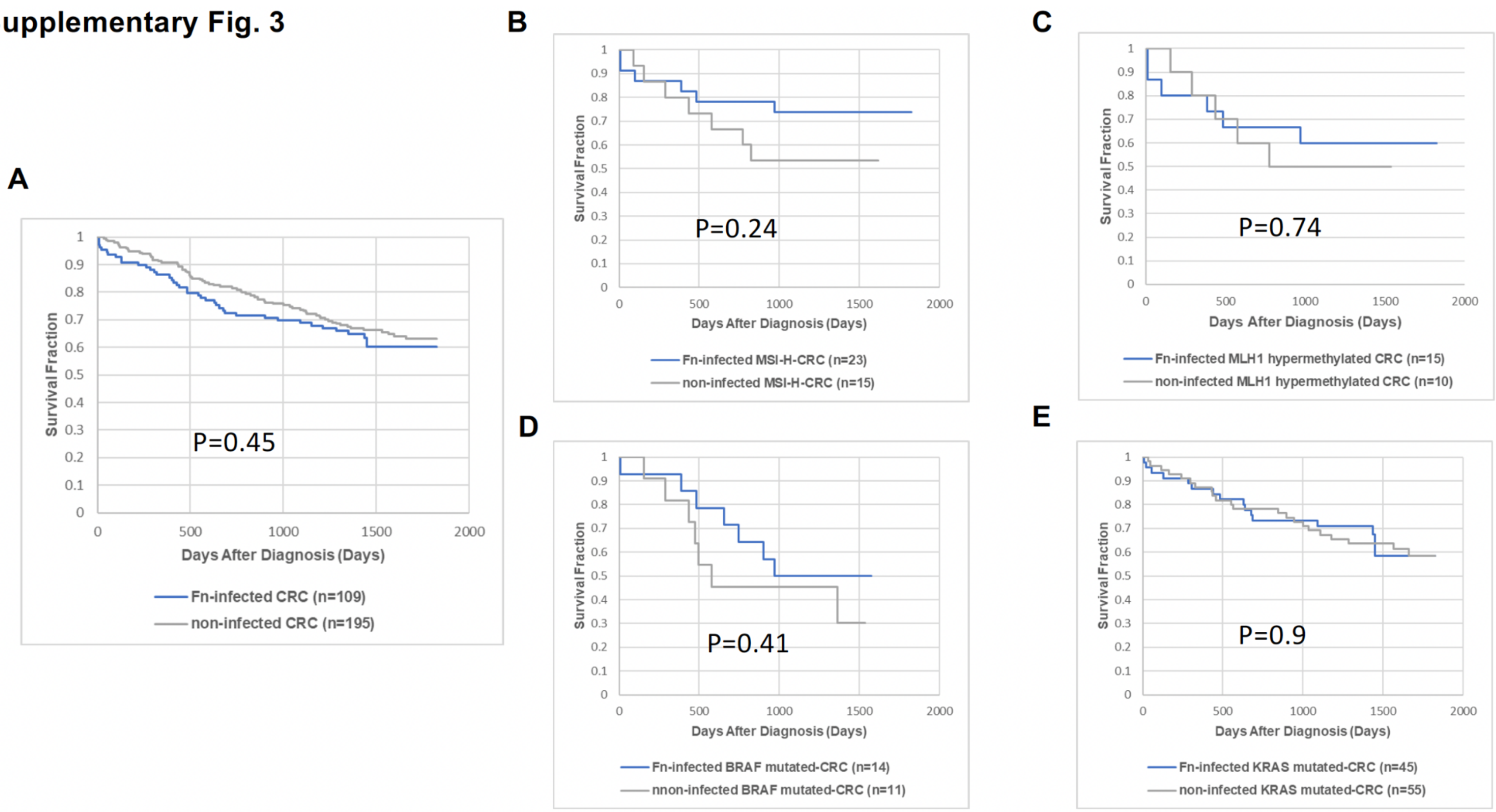

Supplementary Fig. 3: Kaplan-Meier analysis for 5-year overall survival (OS) in CRC patients. (A) Comparison of 5-year OS rates between patients who carried *Fn*-infected CRC (n=109) versus non-infected CRC (n=195). (p=0.45), (B) Between patients who were *Fn*-infected MSI-H-CRC (n=23) and non-infected MSI-H-CRC (n=15) (p=0.24), (C) between patients who carried *Fn*-infected *MLH1* hypermethylated CRC (n=15) and non-infected *MLH1* hypermethylated CRC (n=10)(p=0.74), (D) between patients who carried *Fn*-infected *BRAF*V600E CRC (n= 14) and non-infected *BRAF*V600E CRC (n=11), and (E) between patients who carried *KRAS*-mutated CRC (n=45) and non-infected *KRAS* mutated-CRC (n=55). P values were determined by log-rank test.
